# Supplementary figures and images for: Risk factors of ectopic pregnancy after in vitro fertilization-embryo transfer in Chinese population: A meta-analysis
Source: PLoS One. 2024 Jan 2;19(1):e0296497. doi: 10.1371/journal.pone.0296497 (PMC10760883; doi:10.1371/journal.pone.0296497)

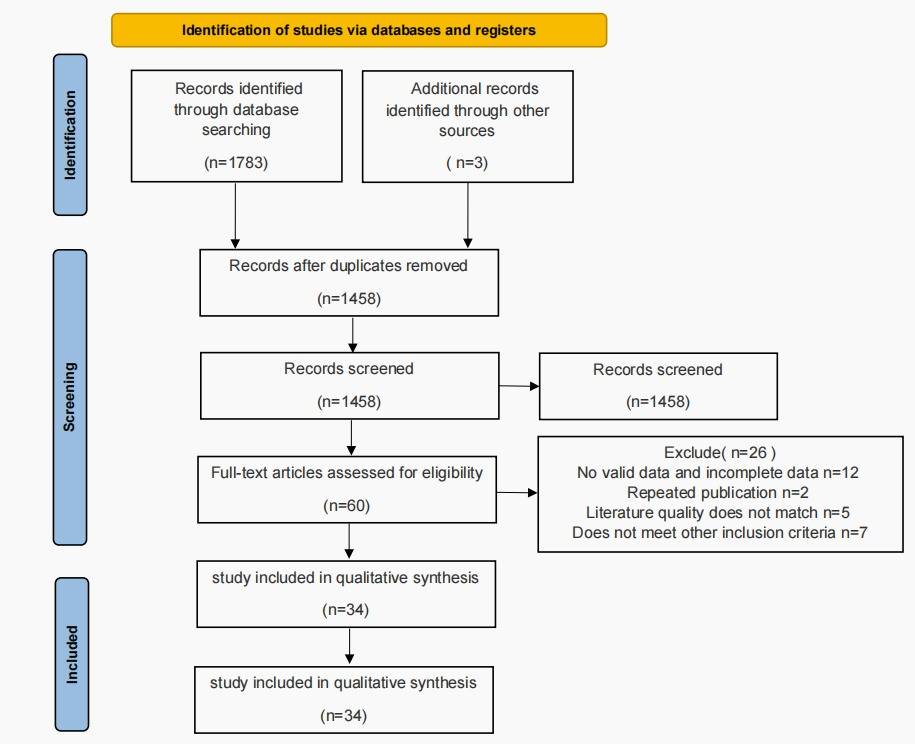

Supplement: S1 Fig — (TIF) [file pone.0296497.s001.tif]

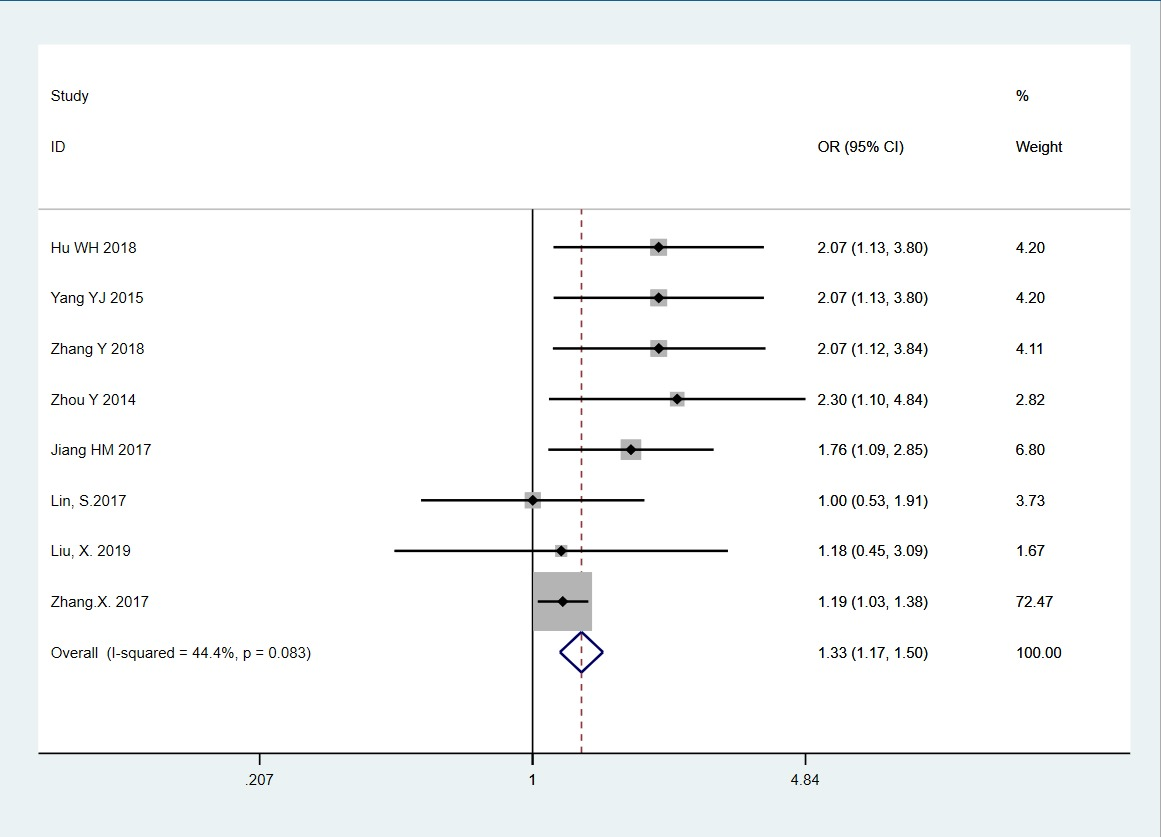

Supplement: S2 Fig — (TIF) [file pone.0296497.s002.tif]
