# Supplementary material for: Risk factors of ectopic pregnancy after in vitro fertilization-embryo transfer in Chinese population: A meta-analysis
Source: PLoS One. 2024 Jan 2;19(1):e0296497. doi: 10.1371/journal.pone.0296497 (PMC10760883; doi:10.1371/journal.pone.0296497)
Supplement: Attachment — Submitted filename: Response to Reviewers.docx [file pone.0296497.s005.docx]

Response to editor

1. Please ensure that your manuscript meets PLOS ONE's style requirements, including those for file naming.

**Response: The format has been revised according to your requirements.**

2. Please update your submission to use the PLOS LaTeX template.

**Response: We have made modifications to the article using the PLOS LaTeX template.**

3. PLOS requires an ORCID iD for the corresponding author in Editorial Manager on papers submitted after December 6th, 2016. Please ensure that you have an ORCID iD and that it is validated in Editorial Manager.

**Response: We have applied for an ORCID ID and verified it in the editor manager. Our 16-digit ORCID identifier is 0009-0009-3585-6339.**

4. Please amend either the abstract on the online submission form (via Edit Submission) or the abstract in the manuscript so that they are identical.

**Response: We have made modifications.**

5. Please include captions for your Supporting Information files at the end of your manuscript, and update any in-text citations to match accordingly.

**Response: We have already done so.**

6. We notice that your supplementary figure is uploaded with the file type 'Figure'. Please amend the file type to 'Supporting Information'. Please ensure that each Supporting Information file has a legend listed in the manuscript after the references list.

**Response: We have made modifications according to your requirements.**

Response to reviewers

1. Is the manuscript technically sound, and do the data support the conclusions?

The manuscript must describe a technically sound piece of scientific research with data that

supports the conclusions. Experiments must have been conducted rigorously, with appropriate

controls, replication, and sample sizes. The conclusions must be drawn appropriately based on

the data presented.

Reviewer #1: Yes

Reviewer #2: Partly

**Response: The statistical results in this meta-analysis support this conclusion.**

2. Has the statistical analysis been performed appropriately and rigorously?

Reviewer #1: Yes

Reviewer #2: I Don't Know

**Response: The statistical methods of this meta-analysis are all operated in strict accordance**

**with the guidelines for meta-analysis.**

3. Have the authors made all data underlying the findings in their manuscript fully available?

The PLOS Data policy requires authors to make all data underlying the findings described in their

manuscript fully available without restriction, with rare exception (please refer to the Data

Availability Statement in the manuscript PDF file). The data should be provided as part of themanuscript or its supporting information, or deposited to a public repository. For example, in

addition to summary statistics, the data points behind means, medians and variance measures

should be available. If there are restrictions on publicly sharing data—e.g. participant privacy or

use of data from a third party—those must be specified.

Reviewer #1: Yes

Reviewer #2: Yes

**Response: We have uploaded my study’s minimal underlying data set as Supporting**

**Information files.**

4. Is the manuscript presented in an intelligible fashion and written in standard English?

   PLOS ONE does not copyedit accepted manuscripts, so the language in submitted articles must be clear, correct, and unambiguous. Any typographical or grammatical errors should be corrected at revision, so please note any specific errors here.

Reviewer #1: No

Reviewer #2: Yes

**Response: According to your request, I have polished my language to a certain extent.**

5. Review Comments to the Author

   Please use the space provided to explain your answers to the questions above. You may also include additional comments for the author, including concerns about dual publication, research ethics, or publication ethics. (Please upload your review as an attachment if it exceeds 20,000 characters)

Reviewer #1: Previous ectopic pregnancy, previous pelvic inflammatory disease, pelvic surgery, anf of course the increase in assisted reproductive techniques are responsibles of the increased incidence of ectopic pregnancies (Tubal and non tubal ectopic pregnancies ex. cesarean scar pregnancy; interstitial, cervical pregnancy, ovarian prengnancy.. ). It is important to clarify the difference between the types of ectopic pregnancies, because they are characterized by extremely different rates or morbidity and mortality ( for example interstitial pregnancy has a mortality rate around 2–2.5%, which is seven times the average for all ectopic pregnancies).
Timely diagnosis is the key for successful and conservative management of ectopic pregnancy.
There is no consensus about the best approach to adopt mainly due to a lack of evidence about the best treatment modality after a comparison in large series of clinical cases or randomized studies. You could explain the different strategies of conservative treatments for the different type of ectopic pregnancies, according to the “objctives” of the study “to provide reference for targeted prevention and treatment”. I think you have to implement this aspect in your discussion.
Section Inclusion /exclusion criteria: Line 58 “same risk factor is greater than or equal to 2 articles. ( 9 )” . You have to put “;” .
Figure 1. You have to correct capital letters
Tables 1. Maybe you can change the format because it is not so clear.
Line 144 “egger 's test “ capital letter.
Lines 162-165 “insufficient ovarian reserve and PCOS are risk factors for ectopic pregnancy after IVF-ET, which may be related to the poor ovarian hormone environment in patients with ovarian dysfunction, and the endocrine support of the ovary to the endometrium is difficult to maintain the best state, resulting in poor endometrial receptivity” Please, explain better.

**Response: According to your requests, we clarified the differences between ectopic pregnancies in the discussion and also added differences in conservative treatment strategies for different types of ectopic pregnancies. We have changed lines 162-165 to “Decreased ovarian reserve and PCOS are also risk factors for EP after IVF-ET. This connection may be related to ovarian endocrine dysfunction and dysregulation of estrogen and progesterone levels in these patients, resulting in poor endometrial receptivity formed by the combined action of estrogen and progesterone”. We have changed the capital letters in Figure 1 and Line 144, and adjusted the format of Table 1.**

**Some of our major modifications:**

**1.We have polished our language to a certain extent.**

**2. At the request of the reviewer, some contents were added to the discussion, and ten**

**references were added.**

**3.We have adjusted the order of authors**
